# The crucial role and mechanism of insulin resistance in metabolic disease

**DOI:** 10.3389/fendo.2023.1149239

**Published:** 2023-03-28

**Authors:** Xuefei Zhao, Xuedong An, Cunqing Yang, Wenjie Sun, Hangyu Ji, Fengmei Lian

**Affiliations:** Guang’anmen Hospital, China Academy of Chinese Medical Sciences, Beijing, China

**Keywords:** insulin resistance, metabolic disease, obesity, T2DM, NAFLD

## Abstract

Insulin resistance (IR) plays a crucial role in the development and progression of metabolism-related diseases such as diabetes, hypertension, tumors, and nonalcoholic fatty liver disease, and provides the basis for a common understanding of these chronic diseases. In this study, we provide a systematic review of the causes, mechanisms, and treatments of IR. The pathogenesis of IR depends on genetics, obesity, age, disease, and drug effects. Mechanistically, any factor leading to abnormalities in the insulin signaling pathway leads to the development of IR in the host, including insulin receptor abnormalities, disturbances in the internal environment (regarding inflammation, hypoxia, lipotoxicity, and immunity), metabolic function of the liver and organelles, and other abnormalities. The available therapeutic strategies for IR are mainly exercise and dietary habit improvement, and chemotherapy based on biguanides and glucagon-like peptide-1, and traditional Chinese medicine treatments (e.g., herbs and acupuncture) can also be helpful. Based on the current understanding of IR mechanisms, there are still some vacancies to follow up and consider, and there is also a need to define more precise biomarkers for different chronic diseases and lifestyle interventions, and to explore natural or synthetic drugs targeting IR treatment. This could enable the treatment of patients with multiple combined metabolic diseases, with the aim of treating the disease holistically to reduce healthcare expenditures and to improve the quality of life of patients to some extent.

## 1 Introduction

More than 100 years have passed since the discovery of insulin, an important regulator of blood sugar, vasodilation, cell growth and protein metabolism. Decreased peripheral target tissue responsiveness to insulin action leads to insulin resistance (IR), a complex pathophysiological condition with reduced sensitivity, impaired ability to inhibit glucose production and stimulate peripheral glucose elimination, and often accompanied with hyperinsulinemia to maintain blood sugar stability (1). IR is characterized by insulin-mediated blood glucose management disorders, blood glucose utilization disorders, abnormal lipid accumulation, and increased lipid decomposition activities in adipocytes, which can be called insulin resistance syndrome or metabolic syndrome. As a hotbed, IR breeds obesity, type 2 diabetes and its complications, non-alcoholic fatty liver disease (NAFLD), tumor, cardiovascular disease and other metabolic diseases. Any disease or disorder that leads to an abnormal metabolic process can be defined as metabolic disease, which poses a major threat to human health and affects the quality of life. Therefore, it is of great necessity to understand IR clearly and explore innovative therapeutic approaches to reduce the burden of disease.

For the diagnosis of IR, the hyperinsulinemic-positive glucose clamp test (HEGC) is considered as the gold standard, but its clinical universality is poor due to its complexity and limitations. There are some less invasive approximations can be used to measure IR, including quantitative insulin sensitivity test index, homeostatic model assessment (HOMA), fasting insulin test, insulin release test, and oral glucose tolerance test (2). Moreover, in clinical and epidemiological studies, the measurement of blood glucose, insulin, and adipokine levels has replaced HEGC for the evaluation of IR (3); for example, elevated levels of branched-chain amino acids and reduced levels of glycine are currently more reliable amino acid markers for IR (4). And some other biomarkers closely related to obesity and metabolic syndrome components, such as adiponectin, fetuin-A and Peptidase M20 domain containing 1 (PM20D1) measurement of their serum concentrations may be valuable for clinical diagnosis of IR-related metabolic and cardiovascular diseases (5–7).

The prevalence of IR and metabolic syndrome is commonly thought to be associated with obesity and T2DM, which inflicts nearly one-third of the world’s population (8, 9). Since IR plays a crucial role in many serious chronic diseases such as type 2 diabetes, cardiovascular and cerebrovascular diseases, the subsequent rise in the incidence of these diseases has made them a major cause of mortality and morbidity worldwide. These metabolism-related diseases not only cause psychological and physical distress to patients, but also place a tremendous burden on health systems, with the total cost, including medical costs and potential loss of economic activity, running into trillions of dollars (8). And patients with metabolic underlying diseases are more vulnerable than healthy individuals in the face of epidemic disease; for example, up to 50% of those who die from COVID-19 have metabolic and vascular disease (10). The increasing incidence of IR and metabolic diseases and the toll they take has prompted an in-depth study of the mechanisms involved. Furthermore, because all these metabolic diseases, as well as IR and obesity, are interrelated through complex molecular-biochemical and immune-related mechanisms, it has been found that many patients encountered in clinical practice today have a combination of multiple metabolic diseases. They interact in a causal manner, with the onset or exacerbation of one disease also affecting other metabolic diseases. For example, among patients with T2DM, the global prevalence of NAFLD is 55.5% (95% CI 47.3-63.7) (11) and up to 50% of hypertensive patients have NAFLD (12). Therefore, if the mechanisms by which IR and metabolic diseases act together can be recognized, so that metabolic diseases with the same mechanisms can be treated with drugs that target IR, this could improve multiple problems for patients and reduce the economic burden. This is crucial for understanding and developing new therapies for many chronic diseases, such as tapping into drugs with multi-target therapeutic effects like metformin, which has good clinical guidance.

The aim of this review is to focus on the key role of IR in a variety of metabolic diseases at multiple levels, including etiology, mechanisms and therapeutic approaches. Moreover, we summarize some of the most recent advances on the pathogenesis and mechanisms of IR. In addition, we outline the available methods for the treatment of IR in terms of non-pharmacological treatment and chemotherapy.

## 2 IR and metabolic disease

**Figure 1:**
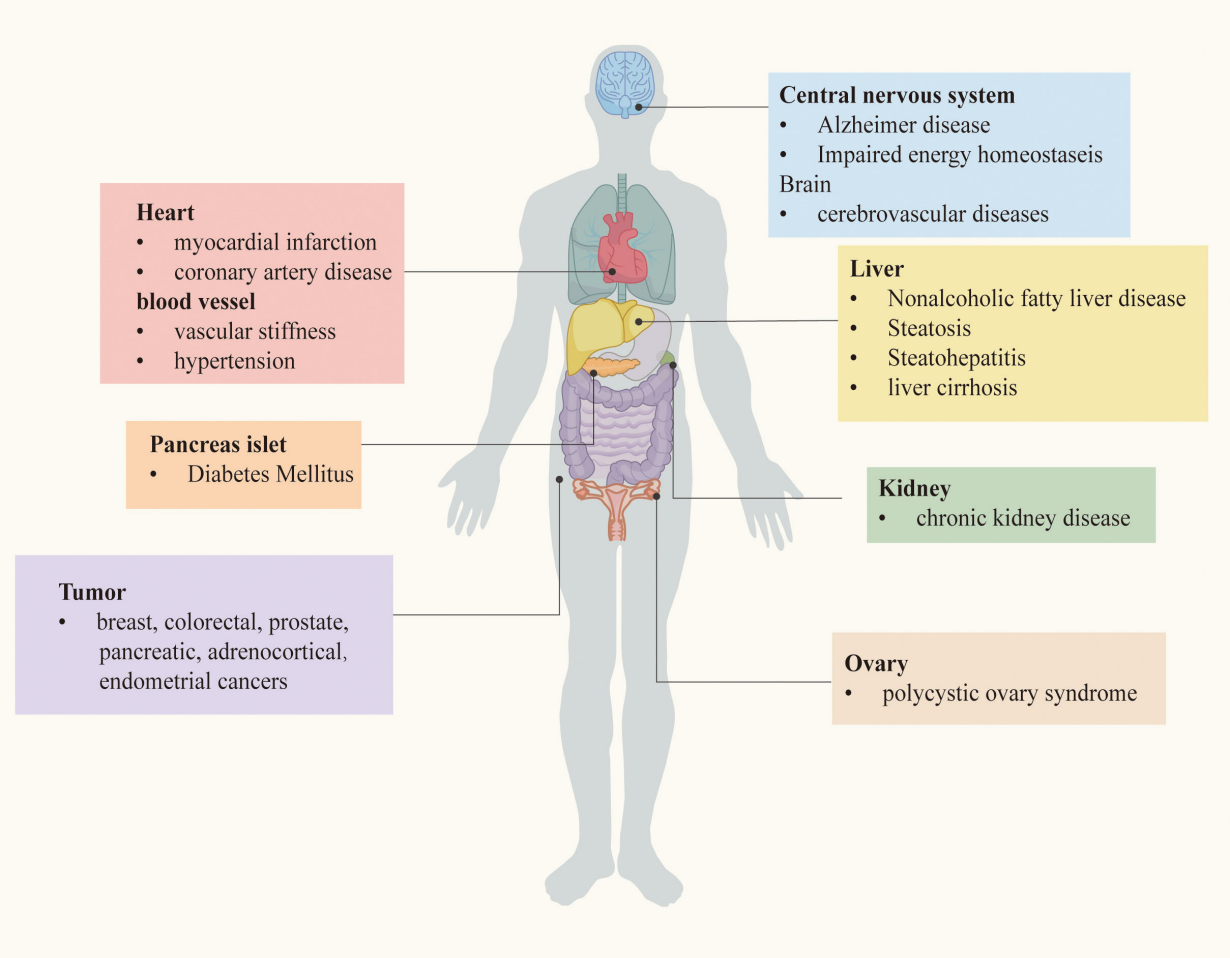
Chronic metabolic diseases may be induced by IR.

As mentioned before, IR is a powerful risk factor for the occurrence and development of a bunch of serious chronic diseases. The relationship between IR and these diseases will be described in turn, based on the clinical researches. Chronic metabolic diseases that may be induced by IR are demonstrated in **Figure 1**.

### 2.1 IR and diabetes mellitus

According to the 10th edition of the IDF Diabetes Atlas, 536.6 million people worldwide have diabetes, which means that more than 10.5% of the world’s adult population has the disease, and this number continues to grow, predicted to rise to 12.2% (783.2 million people) by 2045 (13). Since insulin is a pivotal hormone that regulates blood sugar, IR is closely associated with all stages of DM, including prediabetes, diabetes, and its complications. Impaired β-cell compensation in response to increased IR is a pathophysiological factor associated with poor glucose tolerance, which contributes to the development of DM.

Type 1 DM (T1DM) is caused by the primary loss of β-cells — the cells that release insulin — and the complex autoimmune process of continuous insulin deficiency. Nevertheless, clinical and experimental evidence have shown that patients with T1DM exhibit IR (14), which is a prominent feature in adolescents and adults (15–17), mainly involving the liver, peripheral, and adipose tissue (18). Insulin injections are currently the conventional treatment for T1DM, and prolonged overexposure to insulin itself is a trigger for insulin resistance. patients with T1DM eventually also develop insulin resistance and other features of T2DM, such as cardiovascular disease (19).

Type 2 DM (T2DM) is characterized by defective insulin secretion from pancreatic beta cells. Under normal conditions, increased insulin release by pancreatic β-cells is sufficient of insulin action and maintain normal glucose tolerance (20). However, under the circumstances of IR combined with environmental factors and genetic factors related to T2D, persistent overnutrition sets up a vicious spiral of hyperinsulinemia and insulin resistance, ultimately leading to beta cell failure, possibly due to glucose and lipid toxicity and other factors leading to significant T2D (21). There is a lot of evidence suggesting that both IR and T2D are associated with obesity, especially with high proportion of intra-abdominal and intra-hepatic fat, which is the most crucial factor contributes to the emergence of metabolic disease (22, 23). IR at the beta-cell level may play a role in the pathogenesis of insulin release defects. Reduced insulin release may impair adipocyte metabolism, leading to increased lipolysis and elevated levels of non-esterified fatty acid (NEFA). Elevation of NEFA and glucose can work together to impair islet health and insulin action. Therefore, this process may slowly progress forward to develop T2D (22).

In addition, IR was independently associated with each of the chronic macrovascular and microvascular complications from diabetes (24). Triglyceride-glucose index (TyG index) is a convenient measure of IR. In a large Chinese inpatient cohort study, inpatients with elevated TyG index were shown to be at higher risk for lower extremity macrovascular stenosis, arterial stiffness and renal microvascular injury (25, 26). In particular, IR or hyperinsulinemia is responsible for the development of diabetic cardiomyopathy by pathophysiological mechanisms including impaired insulin signaling, cardiac mitochondrial dysfunction, endoplasmic reticulum stress, impaired autophagy, impaired myocardial calcium handling, abnormal coronary microcirculation, inappropriate neurohumoral activation and maladaptive immune responses (27, 28). Regarding chronic kidney disease, although this remains to be proven, IR is considered to be a factor contributing to the development and progression of diabetic nephropathy (DN), as well as a consequence of DN. IR is exacerbated during the development of DN, possibly due to some potentially modifiable changes in circulating hormones, neuroendocrine pathways, and chronic inflammation (29).

### 2.2 IR and tumor

In recent years, a wealth of experimental, epidemiological and clinical evidence has suggested that IR and its compensatory hyperinsulinemia have a synergistic relationship with the development and progression of certain types of cancer, including breast, colorectal, prostate, pancreatic, adrenocortical and endometrial cancers (30–32). To put it in perspective, IR and hyperinsulinemia, even in individuals without diabetes, are independently and positively associated with increased mortality from pancreatic cancer (33). Besides, according to a large observational study, breast cancer incidence in women with high HOMA-IR is associated with all-cause mortality, especially in postmenopausal women (34). Although the underlying mechanisms of the association between IR and tumor remain unclear, it may rely on several mechanisms and is not necessarily the same for different types of cancers. It is clear that IR-related factors, including chronic persistent hyperinsulinemia, INSRs, IGF1Rs and INSR/IGF1R hybrids, as well as chronic inflammation, ncRNAs and microbiota, have been suggested as factors that may play a role in all tumor stages (35). In addition, the mitogen-activated protein kinase (MAPK) insulin pathway is the basis of many obesity-related malignancies that control cell growth and mitosis (36), whereas insulin can directly promote cell proliferation and survival *via* the phosphatidylinositol-3-kinase/protein kinase B (PI3K/Akt) and Ras/MAPK pathways (37). On the other hand, IR is closely associated with visceral adipose dysfunction and systemic inflammation, both of which favor creating an environment conducive to tumorigenesis (38, 39). Additionally, epigenetic modifications which are triggered by IR and other environmental factors and chronic disease often involve in oncogenesis, such as DNA methylation, histone modifications, and non-coding RNA (35, 40, 41). In addition to the mechanisms described above, recent studies indicate that gut microbiota may be a contributing factor in the relationship between IR and cancer, due to gut dysbiosis (42). Therefore, increasing knowledge about the role of IR in cancer has important implications for cancer prevention and tumor growth inhibition.

### 2.3 IR and cardiovascular and cerebrovascular diseases

IR is thought to be a key risk factor leading to cardiovascular and cerebrovascular diseases in different populations, whether normal or diabetic (43–46). The results of a mathematic analysis indicate that IR, which is responsible for approximately 42% of myocardial infarctions, is probably the most important single cause of coronary artery disease (CAD) (47). Another research showed that compared with patients with lower value of HOMA-IR, those with high value HOMA-IR (≥4.14) had significantly lower global longitudinal strain (GLS), vascular stiffness, and increased pulse wave velocity (PWV) measured in the carotid artery, which have implications for myocardial and vascular function (48). Increased plasma levels of fatty acids in patients with IR and dyslipidemia, with or without diabetes, may lead to the development of metabolism-related cardiomyopathy (49). An example is diabetic cardiomyopathy, which is characterized by diastolic dysfunction and left ventricular hypertrophy in the absence of vascular defects. Diabetic dyslipidemia and lipid accumulation in the myocardium are key pathologic features (50). In animal experiments, mice have shown that when IR develops, insulin receptor substrate-1 (IRS1) and insulin receptor substrate-2 (IRS2) signaling will be impaired, resulting in impaired expression of cardiac energy metabolism genes and activation of p38α mitogen-activated protein kinase (p38), ultimately leading to abnormal cardiac function (51). The strong association between IR and CVD may be due to the fact that the heart is a target organ for insulin, which requires greater energy consumption, yet when IR occurs, it impedes the normal function of the heart and increases the incidence of CVD (52, 53). What’s more, the higher relative risk of cardiovascular events in male with IR compared to female, especially younger women, can be explained by the attenuated relationship between IR and CVD risk (54, 55). Therefore, improving insulin sensitivity not only reduces plasma glucose concentrations in patients with T2DM, but also reduces the risk of cerebrovascular disease independent of the control of blood glucose levels (43, 56).

### 2.4 IR and nonalcoholic fatty liver disease

The liver is one of the main organs controlling the metabolic balance and there is a close relationship between IR and NAFLD, which could be described as a two-way street (57, 58). NAFLD is characterized by excessive accumulation of lipids in hepatocytes. Lipids and metabolites secreted by the liver, including lipoproteins, ketones, acylcarnitine and bile acids, may act as signaling molecules and regulate insulin action (59, 60). Hyperinsulinemia can drive hepatic lipogenesis and lipid accumulation directly as well as through indirect mechanisms, including excess circulating FFA, that impede the ability of insulin to inhibit hepatic glucose production (61). High IR was found to be the most important predictor of NAFLD in both obese and lean subjects (62), and studies have shown that serum insulin levels are strongly associated with hepatic lobular inflammation and histological progression such as ballooning (63). Similarly, in patients with NAFLD, glycerol appearance and lipid oxidation were markedly increased, and IR also increased with the degree of steatosis (64, 65). A meta-analysis showed that compared with those without NAFLD, the risk of T2DM was more than two times higher in patients with NAFLD, with the highest risk particularly in patients with nonalcoholic steatohepatitis (NASH) (66). In the condition of mildly active hepatic steatosis, IR is associated with hepatocellular injury and atherosclerotic dyslipidemia. While in steatohepatitis, IR is combined with cytokine pro-inflammatory status and fibrosis indicators (67).

### 2.5 IR and polycystic ovary syndrome

PCOS is a complex gynecologic endocrine disease, which is characterized by hyperandrogenism, menoxenia, ovulatory dysfunction and infertility. A study of obese adolescent girls indicates that the PCOS phenotype with high androgen levels has the greatest degree of insulin resistance and inflammation (68). Although the etiology and pathogenesis behind PCOS remain to be determined, IR and its compensatory hyperinsulinemia is considered to be an important pathological change that led to progression of PCOS and the main pathological basis for its reproductive dysfunction (69–71). Excessive insulin secretion triggers insulin receptors in the pituitary gland, promoting androgen secretion from the ovaries and adrenal glands through the pituitary-ovary and adrenal axes, and increases free testosterone levels by inhibiting hepatic sex binding globulin (SHBG) synthesis (72, 73). Moreover, insulin, as a reproductive as well as metabolic hormone, has direct effect of stimulating ovarian androgen production by stimulating 17α-hydroxylase activity in the ovarian theca cells and enhance the activity of insulin-like growth factor-1 (IGF-1) receptor in the ovary, thus increasing its free IGF level and promoting androgen production (74, 75). Also, IR has long-term and deleterious effects on the metabolism of women with polycystic ovary syndrome. Irrespective of obesity, 50% of patients with PCOS develop IR (76, 77).

### 2.6 IR and other diseases

In addition to the diseases described above, IR is also associated with many other diseases of various systems throughout the body. This includes liver cirrhosis, which is associated with changes in glucose homeostasis, even in intact liver function. Essential features of the association between cirrhosis and IR include endocrine dysregulation, liver inflammation, changes in muscle mass and composition, changes in the gut microbiota, and permeability (78). IR may also affect the association between insulinemia and bone mass, and Yi-Hsiu Fu et al. found an increased risk of osteoporosis when HOMA-β≥100 and HOMA-IR≥2 in a diverse population (79). Additionally, IR is a crucial risk factor for deterioration of renal function in non-diabetic chronic kidney disease (CKD) and hypertension (80). We also noted the effect of IR in the studies related to postburn trauma (81), postadolescent acne (82), gastro-esophageal reflux disease (GERD) (83) and other diseases.

## 3 Pathogeny of IR

The pathogenesis of IR is the result of the interaction of environmental and genetic factors. Its mechanism of development mainly includes abnormalities in the internal environment, such as inflammation, hypoxia, lipotoxicity, immune environment abnormalities, and abnormal metabolic functions, including metabolic tissues and metabolites.

### 3.1 Heredity

IR and metabolic disorders are commonly clustered in families, which is thought to be the result of an interaction of environmental and genetic factors, although the full genetic background of these conditions remains incomplete (84, 85). Genetic factors associated with IR can be classified as abnormal structure of insulin, genetic defects in the insulin signaling system, genetic defects related to substance metabolism, and other related genetic defects. Mutations in certain insulin-related genes produce mutant human insulins, including Chicago insulin (F25BL*), Los Angeles insulin (F25BS) and Wakayama insulin (V3AL), which have been shown to have significantly reduced insulin bioactivity and decreased binding affinity to the insulin receptor, with consequent effects on insulin sensitivity (86, 87). There are also rare mutations in insulin receptor genes leading to reduced number of cell surface receptors and defective insulin receptor pathways causing hereditary IR, which are found in patients with genetic syndromes of severe IR, such as type A syndrome of extreme IR, leprechaunism, Rabson-Mendenhall syndrome and Donohue syndrome (88, 89). More importantly, since many molecular pathways are involved in energy homeostasis and metabolism, IR is the result of a certain number of mutations in multiple genes, such as those related to type 4 glucose transporter (GLUT4), glucokinase, and Peroxisome proliferator-activated receptor (PPAR) nuclear receptor family, among others (90, 91). Mutations in lipid metabolic pathways, such as mutations in adipocyte-derived hormones such as leptin, adiponectin, resistin or their receptors, mutations in peroxisome proliferator-activated receptors α, γ, and δ, mutations in the lipoprotein lipase gene, and other mutations in genes related to adipose tissue formation can affect the development of glycolipid metabolism and IR (92). For example, the mutation of AKT2/PKBβ in cultured cells may disrupt insulin signaling and inhibit AKT/PKB co-expression (93). The latest advances in high-throughput genetics have revealed the relationship between protein tyrosine phosphatase N1 (PTPN1) and IR, and that the association is mediated by differences in DNA sequences outside the coding region of PTPN1 (94). Healthy carriers of the T allele of TCF7L2 rs7903146, may increase insulin secretion and lead to impaired β-cell function, which is associated with an increased risk of T2DM (95).

### 3.2 Environment

#### 3.2.1 Obesity

Obesity-induced IR is characterized by impaired insulin function that inhibits hepatic glucose output and promotes glucose uptake in adipose tissue and muscle (96). It has been found that weight loss/gain could increase/decrease insulin sensitivity, and obesity and insulin resistance are causally related (97). Using the insulin clamp test, the sensitivity of tissues to insulin decreased by 30% to 40% when body weight exceeded 35% to 40% of ideal body weight. It has been found that waist circumference is closely related to IR, and an increase in waist circumference corresponds to a decrease in glucose consumption or an increase in IR. Conversely, a 10% reduction in BMI improved IR in patients with obesity and T2DM (98). Hence, obesity, especially central obesity, may induce the development of IR due to the massive accumulation of adipose tissue inducing systemic insulin resistance, including endocrine dysregulation and inflammation (99). In obese individuals, especially in those with abdominal obesity, the increase in adipose tissue tends to be more lipolytic, resulting in higher plasma free fatty acid (FFA) levels and intracellular lipid accumulation. Elevated FFA can enhance the phosphorylation of serine residues of insulin receptor substrate (IRS) by activating a series of protein kinases such as c-Jun N-terminal kinase (JNK), whose activity is abnormally increased in obese patients (100, 101). Another mechanism linking obesity and IR is chronic inflammatory responses, including increased production and release of pro-inflammatory factors such as TNF-α, IL-6, and C-reactive protein, which cause insulin resistance in liver, skeletal muscle, and adipose tissue through insulin-interfering signaling pathways (102).

#### 3.2.2 The effect of diseases and drugs

Several physiopathological factors and therapeutic causes, such as chronic hyperglycemia, high free fatty acidemia, certain drugs, such as glucocorticoids, pregnancy, and increased insulin-antagonistic hormones in the body all contribute to the occurrence of IR. There is a pathophysiological relationship between chronic obstructive pulmonary disease (COPD) and IR, partly because the two conditions share common risk factors, such as smoking and lack of physical activity. In addition, systemic effects (deterioration of physical inactivity and sedentary behavior, inflammation) and corticosteroid therapy in patients with COPD may also play a role (103). Also, IR is a common condition after organ transplantation, which leads to new-onset diabetes and metabolic syndrome after transplantation, and subsequent hyperglycemia may significantly increase the morbidity and mortality of cardiovascular disease after kidney transplantation (104, 105). This is due to post-transplant treatment with immunosuppressive agents such as sirolimus, cyclosporine, steroids, etc., leading to IR and metabolic complications (106, 107). In both rodents and humans, exogenous synthetic glucocorticoids such as prednisolone and dexamethasone may induce a number of adverse effects when administered in excess or for prolonged periods, including the development of glucose intolerance, islet-cell dysfunction, IR, hyperglycemia, and dyslipidemia (108, 109). In contrast, almost all morphophysiological changes induced by dexamethasone in the endocrine pancreas are reversed after cessation of treatment (110). Statins may increase IR in peripheral tissues by impairing insulin sensitivity and islet β-cell secretion after long-term use, as it impairs Ca2+ signaling in pancreatic β-cells and downregulates GLUT4 in adipocytes (111).

#### 3.2.3 Aging

Advanced age is an important factor in increasing susceptibility to IR. With increasing age, there is insufficient insulin secretion and a progressive decrease in glucose tolerance, as well as increasing IR due to sarcopenia, excess adiposity and osteoporosis (112, 113). According to epidemiology, the prevalence of IR and T2DM is high in the elderly population (112, 114). This is associated with an increased prevalence of central obesity and increased visceral fat in the aging population (99, 115). In addition to this, factors that increase the risk of IR in the elderly are free radicals that contribute to oxidative stress in old age, and mitochondrial dysfunction (115–117). The paper by Petersen et al. published in the journal Science mentions that older subjects clearly showed reduced insulin-stimulated muscle glucose metabolism compared to younger subjects. To explore the reasons for this, the investigators found increased fat accumulation in muscle and liver tissue as assessed by 1H NMR spectroscopy and an approximately 40% reduction in mitochondrial oxidative and phosphorylation activity as assessed by *in vivo* 13C/31P NMR spectroscopy (118). According to the result of an animal experiment, compared with young mice, aged mice are more susceptible to IR, due to reduced levels of glycolytic proteins and reduced flexible to diet, caused by reduced mitochondrial β-oxidation capacity (119). However, these hypotheses still need to be further tested and further understanding of the metabolic changes associated with aging.

## 4 The mechanism of IR

**Figure 2:**
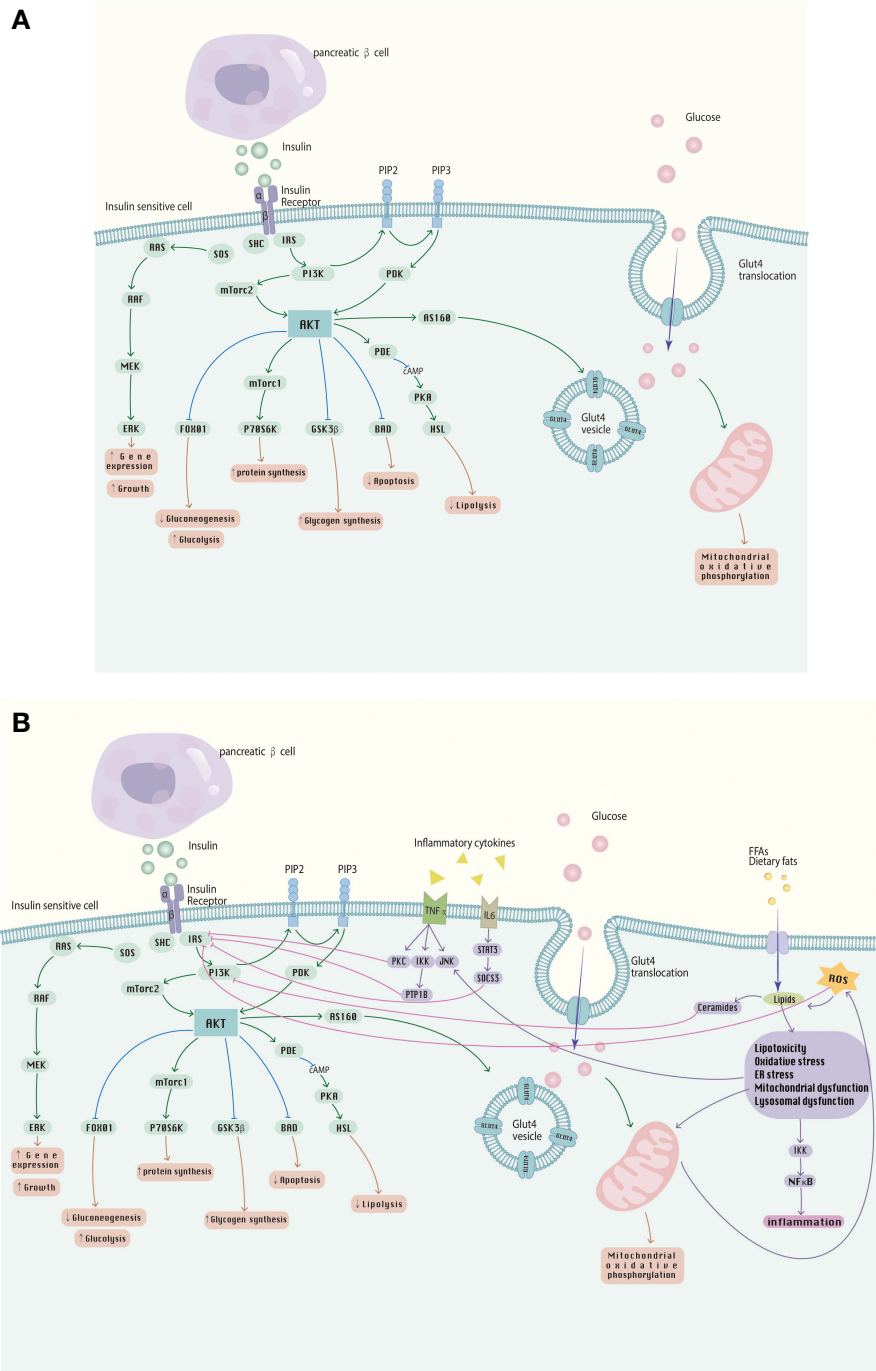
**(A)** The insulin signaling pathway; **(B)** Abnormalities in the insulin signaling pathway caused by inflammatory cytokines, FFA, etc., involved in the development of IR.

The balance of insulin action involves multiple processes in several glucose-utilizing organs or organs, including the liver, adipose tissue, skeletal muscle and kidneys. These metabolic processes receive complex signal regulation. The etiology and pathogenesis of IR are complicated, and the main pathological mechanisms include abnormalities in receptor binding, environment inside the host, intracellular factors, autophagy and intestinal microecology. It is noteworthy that the mechanisms of IR occur somewhat differently in different insulin receptor tissues, and IR appears in a different order, where the initial appearance of IR is in adipose tissue. However, they interact with each other and may eventually develop into systemic IR, a phenomenon verified in observational studies in humans (120–122). In-depth study of the pathogenesis of IR and multiple research directions have become the key to solving the challenges of IR and its related metabolic diseases today. The effects of insulin signaling pathways and the effects of inflammatory cytokines and FFA on them are shown in **Figure 2**.

### 4.1 Insulin receptor defects

Insulin receptors (INSR) which is a tyrosine kinase, bind specifically to insulin and play a key role in insulin-mediated glucose homeostasis and cell growth (123, 124). Impaired INSR binding mainly refers to a decrease in the affinity and number of target receptors on the cell membrane or structural abnormalities of the target receptors that affect insulin binding to the receptor (125). There are several, albeit rare, severe diseases of insulin resistance, including leprechaun’s disease, Rabson-Mendenhall syndrome, or type A insulin resistance syndrome, where insulin binding is severely reduced due to mutations in the insulin receptor gene (88). The insulin receptor substrate protein is generally considered a node in the insulin signaling system, which is closely related to the development of insulin insensitivity. At the molecular level, the crosstalk between the downstream nucleotide-binding oligomerization domain (NOD) 1 effector and the insulin receptor pathway may inhibit insulin signaling by reducing the action of insulin receptor substrates (126). Insulin activates insulin receptor tyrosine kinases, which are capable of aggregating and phosphorylating various substrate docking proteins, such as the insulin receptor substrate (IRS) protein family. Of the four mammalian IRS proteins (IRS-1, IRS-2, IRS-3, IRS-4), IRS1 and IRS2 play key roles in regulating growth and survival, metabolism and aging. They are key substrates of insulin signaling and play an important role in insulin signaling by binding to PI3K and inducing downstream pathways. At the molecular level, dysregulation of the signaling pathway by insulin receptor substrates (IRS) is one of the most common causes of this disease. The current data suggest that insulin-stimulated kinases mediate feedback serine/threonine residues phosphorylation in the IRS and desensitization of proximal insulin signaling plays an important role in the pathogenesis of IR (127). For example the double-stranded RNA-dependent protein kinase (PKR) has also been shown to upregulate the inhibitory phosphorylation of IRS1 and the expression of IRS2 in liver and muscle cells, thereby regulating the insulin signaling pathway. Mediated by two other protein kinases, JNK and IKK, PKR upregulated the phosphorylation of IRS1 at Ser312 and inhibited the tyrosine phosphorylation of IRS1 (128, 129). IRS1 has also been shown to be a target of ceramide-induced Pbx regulating protein 1 (Prep1) and p160 in muscle cells, and the Prep1-p160 axis also affects IRS-1 stability (130). In addition, protein tyrosine phosphatase 1B (PTP-1B), protein kinase C (PKC) and tyrosine residue receptor phosphorylation levels are involved in the regulation of receptor-insulin binding in target tissues. It has been shown that inhibition of PTP1B, a main negative regulator of insulin receptor signaling, can improve glucose homeostasis and insulin signaling (131). In the insulin receptor signaling cascade, protein tyrosine kinase amplifies the insulin signaling response, and phosphatase is necessary to regulate the rate and duration of the reaction (132).

IR occurs in a variety of tissues, including skeletal muscle, liver, kidney and adipose tissue, and its mechanisms are specific. Among the target organs of insulin, bone, as an endocrine organ, can regulate energy homeostasis by altering insulin sensitivity, dietary behavior, and adipocytes (133). There seems to be a bilateral relationship between bone and IR that binds them together in a biological partnership (134). Among them, skeletal muscle estrogen receptor α plays a crucial role in maintaining systemic glucose homeostasis and insulin sensitivity (135). Beyond that, skeletal muscle is an important insulin-sensitive tissue, accounting for approximately 80% of insulin-dependent glucose uptake. It has been repeatedly demonstrated that skeletal muscle tissue plays an important role in the maintenance of systemic glucose homeostasis and overall metabolic health. In addition, the crosstalk between muscle factors and adipokines leads to negative feedback, which in turn aggravates muscle reduction obesity and IR (136). In the kidney, the effector cells of insulin are podocytes in which nucleotide-binding oligomerization domain 2 (NOD2) is highly expressed. NOD2 is a major member of the NOD receptor family and is involved in the innate immune response. It induces podocyte IR by activating the inflammatory response (137). In terms of hepatic IR, IRA, one of the isoforms of the insulin receptor, whose expression in the liver of mice on a high-fat diet increase hepatic glucose uptake, decrease lipid accumulation, and reduce or at least delay the development of fatty liver and NASH. This suggests that a gene therapy approach to hepatic IRA expression could act as a facilitator of glucose uptake in IR states (138–140).

### 4.2 Abnormal insulin signaling

Insulin acts by binding to the INSR and activating downstream signaling pathways which have been extensively studied. After binding to INSR, insulin acts mainly through two major signaling pathways, the phosphatidylinositol 3-kinase (PI3K)–serine-threonine kinase (AKT)/protein kinase B (PKB) pathway, which plays a part in regulating metabolism, and the Ras–mitogen-activated protein kinase (MAPK) pathway, which is mainly responsible for controlling cell growth and differentiation (141). Although where the defect occurs in the insulin signaling pathway remains a matter of doubt, many key insulin signaling pathway components have been identified. These components can be divided into proximal components, including insulin receptors, insulin receptor substrates, PI3K, and AKT/PKB, and distal components, representing various components downstream of AKT/PKB, including TBC1D4, GSK3, and PDE3B. IR is caused by defects in one or more of these signaling components (142).

### 4.3 Abnormal internal environment

Environment, such as diet and exercise, and genetics, as well as the interaction between the two, play a major role in the development of IR and metabolic disease. Exercise and dietary habits may directly or indirectly drive changes in the host internal microenvironment. Current research suggests that extracellular influences such as inflammation, hypoxic environments, lipotoxicity or immune abnormalities can trigger intracellular stress in key metabolic target tissues, which impairs the normal metabolic function of insulin in these cells thereby causing the progression of whole-body IR (143).

#### 4.3.1 Inflammation

Obesity characterized by a chronic, low-grade inflammatory state is closely associated with IR. In the obese state induced by diet, there is a significant increase in lipid accumulation and increased secretion of pro-inflammatory cytokines by adipocytes and macrophages, including pro-inflammatory response factors such as tumor necrosis factor‐α (TNF-α), monocyte chemotactic protein‐1 (MCP‐1) and interleukins (IL), as well as increased production and release of C-reactive protein (CRP), which induce insulin resistance through multiple mechanisms, including activation of Ser/Thr kinases, decreasing IRS-1, GLUT4, and PPARγ expression, or activation of SOCS3 in adipocytes (99, 144–146).

##### 4.3.1.1 Inflammatory factors

The mechanisms of inflammation leading to IR mainly include inflammatory factors acting on the insulin signaling system to interfere with INSR signal transduction. TNF-α and IL-1β are additional macrophage-derived pro-inflammatory mediators that directly affect insulin sensitivity (147, 148). TNF-α stimulates insulin-resistant adipose tissue through IRS protein interference by abnormal signals on phosphorylated serine residues of IRS1 (149). In addition, TNF-α could affect insulin signaling through serine phosphorylation and kinase pathway defects (99, 150). CRP is another marker of inflammation associated with IR and metabolic diseases and is a widely used clinical biomarker. CRP binds to leptin, blocks leptin signaling and modulates its central action and hypothalamic signaling, thereby directly interfering with energy homeostasis, insulin sensitivity and glucose homeostasis (151, 152).

##### 4.3.1.2 Inflammatory pathway

Furthermore, the activity of signaling molecules in inflammatory pathways such as IκBα kinase β (IKKβ)/nuclear factor-kappaB (NF-κB) and JNK1 was found to be activated in adipose tissue and liver (100, 153). The above pro-inflammatory cytokines exert their effects by stimulating major intracellular inflammatory pathways, and the activation of these pathways also promotes increased expression of the inflammatory factors involved in IR. Toll-like receptor (TLR), especially TLR4, participates in IR-related inflammation by increasing the gene expression of IKKβ, NF-κB transcription factors, and pro-inflammatory mediators in adipose tissue macrophages (154–157). IKK is an enzyme complex that activates the NF-κB transcription factor (144). It has also been shown that NF-κB receptor activator (RANKL) is a potent stimulator of NF-κB and that systemic or hepatic blockade of RANKL signaling leads to significant improvements in hepatic insulin sensitivity and prevents the development of diabetes (158). On the other hand, metabolic stress activates the JNK signaling pathway, which can inhibit the tyrosine phosphorylation level of IRS in target tissue cell membranes, which in turn affects downstream insulin-related signaling such as PI3K and Akt/PKB, resulting in IR (99, 159). And JNK signaling in adipocytes leads to an increase in circulating concentrations of hepatic factor fibroblast growth factor 21 (FGF21), which regulates systemic metabolism (160).

##### 4.3.1.3 Immunocytes

In the pathogenesis of IR and metabolic diseases, immune cells play a crucial role. Adipose tissue contains most types of immune cells, which under conditions of obesity contribute to a complex network of inflammation and IR with activation and infiltration of pro-inflammatory immune cells in adipose tissue, including macrophages, neutrophils, eosinophils, mast cells, NK cells, MAIT cells, CD4 T cells, CD8 T cells, regulatory T cells and B cells, as well as high levels of pro-inflammatory molecules (161). Among them, adipose tissue macrophages can be divided into M1 phenotype (pro-inflammatory macrophages) and M2 phenotype (anti-inflammatory macrophages), representing the two extremes of macrophage polarization. M1 macrophages are highly antimicrobial and antigen-presenting, producing pro-inflammatory cytokines, such as TNF-α, and reactive oxygen species (ROS) that worsen inflammation, mast cells, neutrophils and dendritic cells directly or indirectly exacerbate IR (162). In contrast, M2 macrophages help maintain insulin sensitivity in lean adipose tissue, as well as eosinophils and innate lymphocytes appear to have a protective effect on glucose homeostasis and insulin sensitivity (163–168). Crosstalk between M1-M2 macrophage polarization plays an important role in IR through the shift from M1 to M2 phenotype and activation of transcription factors (162, 169). Dysregulation of visceral adipose tissue macrophage (ATM) response to microenvironmental changes underlies the development of abnormal local and systemic inflammation and IR (170). In the obese state, enhanced macrophage infiltration and secretion of various inflammatory cytokines in white adipose tissue activate JNK and NF-κB, causing local and systemic IR (171, 172). Macrophages can alter their phenotype in response to changes in the microenvironment and macrophage differentiation.

In the past, more attention has been paid to the regulation of insulin sensitivity by innate immune cells, particularly macrophage mediated, which have been mentioned before. Cells of the adaptive immune system, B lymphocytes and T lymphocytes, and their respective subsets, are also thought to be important regulators of glucose homeostasis and play an important role in the immunopathogenesis of autoimmune diabetes (168, 173, 174). Studies have shown that CD4(+) T lymphocytes in visceral adipose tissue (VAT) control the progression of metabolic abnormalities associated with obesity, including expansion of adiposity and pathogenic VAT T cells, which can be successfully reversed by immunotherapy (175, 176). Impaired through an adaptive immune response, IR can also be driven by inflammation and dysregulation of the gut microbiota, as in pathogen-induced periodontitis (177). In addition, the intestinal immune system is an important regulator of glucose homeostasis and obesity-related IR in turn affects intestinal permeability and thus systemic IR (178). Another essential part of the immune defense system is the complement system. It plays an important role in activating innate and adaptive immune responses, promoting apoptosis, and eliminating damaged endogenous cells. Patients with obesity exhibit activation of the complement system in their adipose tissue, which is connected to changes in glucose metabolism and subclinical inflammation (179).

#### 4.3.2 Hypoxia

Adipose tissue hypoxia is causally related to obesity-induced IR, especially in high-fat diet (HFD) fed and early obese patients, as adipocyte respiration becomes uncoupled, resulting in a state of increased oxygen consumption and relative adipocyte hypoxia (180). Clinically, obstructive sleep apnea (OSA), characterized by intermittent hypoxia (IH), is a widely prevalent respiratory disorder with a particularly high prevalence in obese patients and is associated with IR and metabolic diseases such as hypertension, cardiovascular risk and NAFLD (181, 182). Not only in obese individuals, but an animal study found that IH cause acute IR in lean or healthy mice, which is related to reduced glucose utilization in oxidized muscle fibers. As the glucose infusion rate decreased, hypoxia induced systemic IRA (183). The key regulators of oxygen homeostasis in response to hypoxia are the hypoxia-inducible factors (HIFs), a family of transcription factors activated by hypoxia. Adipocyte hypoxia could trigger HIF-1α induction causing adipose tissue inflammation and IR (180, 184). HIF-1-mediated activation of NOX4 transcription and the consequent increase in H2O2 led to intermittent hypoxia-induced pancreatic β-cell dysfunction (185). In hypoxic adipocytes, HIF-1α activates the NLRP3 inflammasome pathway and stimulates IR by upregulating the expression of pla2g16. In obesity-induced intestinal hypoxia, HIF-2α increases the production of ceramide, to promote the expression of the key enzyme sialidase 3 encoding Neu3, which leads to the development of IR in obese mice induced by a high-fat diet (186). While in skeletal muscle, hypoxia is a stimulus stimulating GLUT4 translocation *via* activation of AMPK, causing defects of glucose transport and this may counteract IR (187).

#### 4.3.3 Lipotoxicity

Insulin regulates lipid metabolism through the typical insulin signaling cascade, while metabolites can also directly regulate insulin sensitivity by modulating components of the insulin signaling pathway (188). Lipids have multiple roles as signaling molecules, metabolic substrates and cell membrane components, and can also alter proteins that affect insulin sensitivity (189). Lipotoxicity is when the storage capacity of adipose tissue is overloaded due to obesity, overnutrition, etc., leading to uncontrolled accumulation of lipids in ectopic non-adipose tissues (e.g., liver, heart, pancreas, and muscle). High concentrations of lipids and lipid derivatives cause deleterious effects on cells through mechanisms including oxidative stress, endoplasmic reticulum (ER) stress, c-Jun NH2-terminal kinase (JNK)-induced toxicity, and BH3-pure protein-induced mitochondrial and lysosomal dysfunction (190, 191). Numerous studies have reported that Adipose tissue dysfunction and lipotoxicity play a role in metabolic disorders and IR (192, 193). This is associated with a chronic elevation of free fatty acids (FFA, also called non-esterified fatty acids) in plasma due to adipose tissue dysfunction (99). Adipose malnutrition or adipose tissue dysfunction can lead to pathologically elevated FFAs. Chronically elevated FFAs appear to cause adipocyte production of inflammatory factors, decreased insulin biosynthesis, glucose-stimulated insulin secretion, and glucose sensitivity in β-cells. The ER stress pathway is a key mediator of inflammation induced by serum excess FFA and IR in various cell types, and PERK and IKKβ are key signaling components (194). The obesity-induced increase in adipocyte volume and tissue mass will lead to inflammation, additional disturbances in adipose tissue function, and ultimately adipose tissue fibrosis (195). Adipose tissue macrophages are an abundant immune component of hypertrophy, which plays a key role in diet-induced T2DM and IR (196).

In renal ectopic lipid accumulation, lipotoxicity promotes podocyte injury, tubular injury, thylakoid proliferation, endothelial cell activation and macrophage-derived foam cell formation, which contribute to the development of renal IR and other renal diseases, especially diabetic nephropathy (197). In skeletal muscle, sustained nutrient overload of L6 myotubes leads to lipotoxicity that promotes activation of the IKKβ-NFkB pathway in muscle cells, inducing increased cellular ROS and impaired insulin action in the myotubes (198). Saturated fatty acids are known to increase the production of lipotoxic products such as ceramide and diacylglycerol, which disrupt islet beta-cell function, vascular reactivity and mitochondrial metabolism, and also play a key role in the induction of muscle IR (199–201). Similarly, defective fatty acid oxidation (FAO) and consequent lipotoxicity in cardiac cells induce a range of pathological responses, including oxidative stress, DNA damage, inflammation and insulin resistance. The obesity-mediated atrial fibrillation and structural remodeling can be attenuated by promoting FAO, activating AMPK signaling and attenuating atrial lipotoxicity through levocarnitine (LCA) (202). Lysophosphatidic acid (LPA) is an effective, biologically active lipid. After binding to G protein-coupled receptors, it can profoundly affect cell signal transduction and function. Metabolic and inflammatory disorders, including obesity and IR, are associated with modifications in LPA signaling as well as the production and function of autocrine motility factors (203). Additionally, it has been discovered that the anti-adipogenic transcription factor GATA-3 is a possible molecular target that affects adipogenesis. Those with obesity and IR exhibit increased GATA-3 expression when compared to insulin-sensitive individuals with BMI matches (204). While lifestyle interventions such as physical activity have been confirmed to have a positive effect on insulin sensitivity in skeletal muscle, affecting lipid metabolism (205).

##### 4.3.3.1 Ceramide accumulation

Ceramides are a family of lipid molecules consisting of sphingosine and a fatty acid. The synthesis of *de novo* ceramides depends on the availability of free fatty acids, especially palmitate, whose over-intake may lead to an excessive accumulation of ceramides (206). In addition to their function in lipid bilayers, these molecules are also thought to be biologically active agents involved in a variety of intracellular pathways, such as free radical production, release of inflammatory cytokines, apoptotic processes, and regulation of gene expression. Ceramides are metabolic products that accumulate in individuals suffering from obesity or dyslipidemia and alter cellular processes in response to fuel overload (207). ceramides accumulation over time modulates signaling and metabolic pathways that drive lipotoxicity and IR, causing tissue dysfunction (208). Numerous studies have been conducted in recent years to confirm the critical role played by ceramides in glucose homeostasis and insulin signaling (199). These evidence are particularly strong in skeletal muscle, while the data in liver and WA are somewhat more equivocal (209, 210). Ceramides are synthesized by ceramide synthase (CerS) through N-acylation. To date, six mammalian CerS have been identified (CerS1-6) that show different affinities for the fatty acid acyl-CoA chain length used for sphingomyelin N-acylation. Among them, CerS1 is most abundant in skeletal muscle and is responsible for the synthesis of C18:0-Cer, which negatively regulates insulin sensitivity in obese and/or T2D subjects (211). CerS2 is the major isomer in the liver that preferentially makes extra-long-chain (C22/C24/C24:1) ceramides, which inhibits β-oxidation, leads to a compensatory increase in long-chain C16-ceramides, and makes one susceptible to diet-induced fatty liver and IR (212). CerS6 is specific for C14 and C16 acyl chain lengths, and CerS6 levels are significantly increased in obese adipose tissue (212, 213). The main mechanism by which ceramides promote IR is through inhibition of proximal insulin signaling components, such as Akt/PKB activity. Ceramide can inhibit Akt/PKB activity by increasing protein phosphatase 2A (PP2A) activity to stimulate Akt/PKB dephosphorylation and blocking Akt/PKB translocation through PKCζ (214). Activation of PP2A inhibits Akt/PKB by impairing serine phosphorylation of Akt/PKB, thereby reducing the transfer of GLUT4 to the plasma membrane and thus reducing glucose uptake (215, 216). In addition, ceramide may cause IR by accumulating in mitochondria and causing mitochondrial reactive oxygen species (ROS) or by promoting the secretion of pro-inflammatory factors (217).

##### 4.3.3.2 Diacylglycerols accumulation

Another lipid metabolite closely associated with IR is DAG, whose accumulation in skeletal muscle, adipocytes and liver is thought to promote IR by altering cellular signaling at its specific location, due to increased serum FFA levels (218). The DAG hypothesis of IR is that the interference of activated PKC, especially the novel PKC isoforms including δ, ϵ, ν, and θ, with insulin signaling is due to the accumulation of DAG in insulin-sensitive tissues (219, 220). In particular, 1,2-DAG, which derives from esterification and accumulates mainly in the membranes, is clearly associated with PKC activation, and these isoforms then phosphorylate IRS1 serine with the result that decrease PI3K activation (211, 221).

It is worth noting that the role of intracellular ceramide and DAG in IR is controversial and that defects in these components are unlikely to be the sole cause of IR. It is true that not all studies have confirmed a role for the DAG-PKC-insulin receptor pathway in IR; for example, some studies have shown that PKCϵ deficiency in the liver has no effect on systemic insulin sensitivity in mice (222), and there are also experiments in which acute knockout of PKCϵ in the liver protects rats from IR (218). Therefore, more in-depth studies on proximal insulin signaling with DAG and ceramide are still needed.

### 4.4 Organelle interaction

Organelles, including the endoplasmic reticulum (ER), mitochondria and endoplasmata, contribute to a range of cellular functions through their unique local environment and molecular composition. Organelles can actively communicate and cooperate with each other through vesicle trafficking pathways and membrane contact points (MCSs) to maintain cellular homeostasis, which facilitates the exchange of metabolites and other information required for normal cellular physiology (223). Imbalances in organelle interactions may lead to various pathological processes, such as imbalances in cellular energy metabolism (224). Recent studies have shown that mitochondria could interact with various organelles (225), which are essential for energy metabolism and cell survival, and increasing evidence shows that mitochondrial dysfunction in skeletal muscle and mitochondrial overactivation may induce IR (226).

The production of mitochondrial ROS is thought to adjust skeletal muscle insulin sensitivity. Mitochondrial quality control mechanisms are regulated by PGC-1α, which may affect age-related mitochondrial dysfunction and insulin sensitivity (227). The continuous processes that occur in the skeletal muscle after excessive intake of a high-fat diet include the accumulation of cytosolic fatty acids, increased production of ROS, mutation, and aging. The ensuing mitochondrial dysfunction could lead to decreased β-oxidation, respiratory function, and increased glycolipid toxicity. Together, these events induce IR in the skeletal muscle (228). The physical contact site between the mitochondria and endoplasmic reticulum (ER) is called the mitochondrial-associated membrane (MAM). The imbalance of MAMs significantly leads to IR. ER stress may be the main mechanism by which MAM induces IR in the brain, especially in the hypothalamus (229, 230). Exosome-like vesicles (ELVs) are the smallest type of extracellular vesicles released from cells that play a role in cell crosstalk because they regulate insulin signaling and β-cell quality, and released ELVs leading to IR or β-cell apoptosis (231).

### 4.5 Other influence elements

#### 4.5.1 Phosphatase and tensin homolog

PTEN is not only a tumor suppressor gene but also a metabolic regulator. Under physiological and T2D conditions, PTEN also has a negative regulatory function in insulin signaling through its inhibition in the PI3K pathway (232, 233). PTEN reduces the level of phosphatidylinositol-3, 4, 5-phosphate (PIP3). This leads to impaired insulin signaling and promotion of IR in the pathogenesis of T2D. The function of PTEN in regulating insulin signaling in different organs has been identified. The role of PTEN in the regulation of insulin action in many cell types has been elucidated through mouse models of lacking PTEN in metabolic organs and *in vitro* cell culture (234, 235). Interventions targeting PTEN regulatory signaling may therefore be a promising target aimed at reversing insulin resistance. Interventions targeting PTEN regulatory signaling may therefore be a promising target aimed at reversing insulin resistance.

#### 4.5.2 Vitamin D

In addition to its effects on skeleton, Vit D has significant effects on pancreatic β-cells function and metabolic syndrome including blood pressure, abdominal obesity, glucose metabolism associated with it, as calcitriol functions as a chemical messenger by interacting with calcium flux-regulating receptors on beta cells (236). As the results of a meta-analysis showed, there was an inverse relationship between serum Vit D concentration and metabolic syndrome risk in the general adult population in cross-sectional studies (237). The molecular mechanisms of Vit D deficiency involved in the development of IR may be because it maintains normal resting levels of ROS and Ca2+ in pancreatic β-cells and also reduces the degree of IR-related pathological degrees, such as oxidative stress and inflammation (238). Vitro studies showed that Vit D could regulate lipid and glucose metabolism in adipose tissue, skeletal muscle and liver, and pancreatic insulin secretion (239).

#### 4.5.3 Minerals

Minerals are essential micronutrients for the human body. Deficiencies in certain micronutrients due to differences in diet composition may lead to imbalances in glucose homeostasis and IR (240).

Magnesium is a cofactor required for glucose access to cells and carbohydrate metabolism, and it has the function of regulating the electrical activity of pancreatic beta cells and insulin secretion (240). Mechanistically explained, magnesium is a cofactor in the downstream action of the insulin cascade. Low magnesium ion levels lead to defective tyrosine kinase activity, blocking intracellular insulin action and altered cellular glucose transport, thus promoting IR (241). On the other hand, magnesium deficiency inhibits cellular defenses against oxidative damage and triggers chronic systemic inflammation that enhances IR. As demonstrated in a longitudinal study, magnesium intake was also inversely associated with high-sensitivity CRP, IL-6 and fibrinogen levels, as well as HOMA-IR (242). There is evidence suggesting that magnesium supplementation attenuates IR in patients with hypomagnesemia-associated IR (243). Also, animal studies have shown that dietary magnesium supplementation to increase plasma magnesium concentrations reduces blood glucose levels, improves mitochondrial function, and reduces oxidative stress in diabetic mice (244). However, new intervention studies are still needed to clarify the role of nutrients in the prevention of this metabolic disorder, as well as to standardize the type, dose, and timing of magnesium supplementation.

Zinc is an essential micronutrient for metabolism, which plays a particularly critical role in the islets. Diabetes affects zinc homeostasis, and disturbances in zinc homeostasis have been associated with diabetes and IR (245). Because zinc is an essential component of insulin, it regulates islet cell secretion and promotes its binding to hepatocyte membranes while maintaining phosphorylation and dephosphorylation levels of the receptor. Zinc influx mediated by Slc39a5, a zinc exporter in pancreatic β-cells, plays a role in insulin processing and secretion by inducing Glut2 expression through Sirt1-mediated activation of Pgc-1α (246). In addition, zinc acts as a pro-antioxidant to reduce the formation of ROS, which is particularly beneficial in aging and IR (247). Mineral deficiencies are directly or indirectly associated with oxidative stress, which ultimately leads to IR or diabetes (240).

### 4.6 Nervous system effects

The brain is also an insulin-sensitive organ with a large number of insulin receptors distributed (248, 249). The action of insulin in the brain produces a variety of behavioral and metabolic effects that influence eating behavior, peripheral metabolism, and cognitive performance (250). Disturbances in the role of insulin in the brain reveal a possible link between metabolism and cognitive health. The hypothalamus plays a fundamental role in the survival and control of physiological processes necessary for vital physical functions, including various endocrine functions. Injecting insulin *via* intranasal administration leads to an increase and subsequent decrease in plasma insulin, affecting peripheral metabolism, and a decrease in BOLD signaling and cerebral blood flow in the hypothalamus is observed (250, 251). It appears that the effects of central insulin may have a biphasic effect on peripheral insulin sensitivity (251). Insulin signaling has been shown to affect the molecular cascade of hippocampal plasticity, learning, and memory (252). Furthermore, the insulin-responsive glucose transporter GluT4 has a key part in hippocampal memory processes, and reduced activation of this transporter may underlie IR-induced cognitive deficits (253).

### 4.7 Autophagy

Autophagy is a self-degrading process that is conserved in all eukaryotic cells and plays a crucial role in balancing energy sources during critical periods of development and in response to nutritional stress. Autophagy also promotes cellular senescence and cell surface antigen presentation, prevents genomic instability and necrosis, and it is an important mechanism for a variety of physiological processes, such as cellular homeostasis, senescence, immunity, oxidation, differentiation, and cell death and survival (254). Recent studies have shown that autophagy is an important regulator of organelle function and insulin signaling, and that loss of autophagy is a key component of defective insulin action in obesity, which may be specifically related to ER function (255). It has been found that autophagy deficiency and its resulting mitochondrial dysfunction increase fibroblast growth factor 21 (Fgf21) expression through the induction of Atf4. The induction of Fgf21 promotes protection against diet-induced obesity and IR (256). In addition, exercise induces autophagy through the regulator BCL2, which may contribute to beneficial metabolic effects and improve IR in muscle (257).

### 4.8 Intestinal microecology

In addition to the aforementioned influences such as metabolites and cytokines, the 100 trillion bacterial colonized gut microbiota can also contribute to IR (220, 258). Patients with metabolic syndrome showed increased insulin sensitivity after six weeks of infusion of gut microbiota from lean individuals. Levels of gut microbiota producing butyrate, which has been shown to prevent and treat diet-induced insulin resistance in mice by promoting energy expenditure and inducing mitochondrial function, were also increased (259, 260). Dietary reasons for obesity may promote IR both through mechanisms independent of the gut microbiota and through mechanisms dependent on the bacterial community (261). Intestinal dysbiosis is associated with the transfer of bacterial lipopolysaccharide (LPS) into the systemic circulation and its induction of metabolic endotoxemia, leading to a chronic subclinical inflammatory process and the development of IR through activation of toll-like receptor 4 (TLR4) (261–263). In addition to the LPA mentioned above, branched-chain amino acids (BCAAs) are another harmful gut microbially regulated metabolite whose levels are increased in the serum metabolome of IR individuals. *Prevotella copri* has been shown in mice experiments to induce IR, exacerbate glucose intolerance and increase circulating levels of BCAAs (264). Moreover, gut microbiota-derived short-chain fatty acids (SCFA) may improve IR and prevent T2DM by reducing the secretion of pro-inflammatory cytokines and chemokines and decreasing local macrophage infiltration, as well as increasing the lipid storage capacity of white adipose tissue (121, 265, 266). Taken together, targeting gut microbes may have the potential to reduce IR and decrease the incidence of related metabolic diseases.

## 5 Treatment of IR

### 5.1 Nondrug treatment

Today’s modern lifestyle is characterized by reduced energy expenditure, consumption of high-calorie junk food and fast food, sedentary lifestyle, irregular eating occasions and eating times especially for late night snacking, and chronic psychological stress. This lifestyle triggers several mechanisms such as the development of IR that aggravate metabolic stress. Studies have shown that lifestyle interventions through small weight loss (7-10%), 150 minutes of moderate intensity exercise per week and behavioral therapy approaches are very effective in preventing and treating IR and T2DM (267). According to the ADA/EASD consensus guidelines, lifestyle recommendations are the first-line therapy, followed by metformin for T2D. Next, the contribution of non-pharmacological therapies, including exercise and diet, to the alleviation of IR will be elaborated.

#### 5.1.1 Physical exercises

Exercise is well known to improve metabolic disease by improving obesity and enhancing insulin sensitivity. A meta-analysis determined the effectiveness of a structured exercise intervention program for IR in T2DM, and the evidence highlights that regular exercise improves glycemic control and therefore can be recommended for reducing IR with a moderate level of evidence (268). As we know, physical exercise increases the oxidative capacity and biogenesis of mitochondrial substrates in skeletal muscle. It was shown that treadmill training modulates the increase in mitochondrial substrate oxidation in liver and skeletal muscle induced by a high-energy diet in mice, disconnecting it from pyruvate and acetyl CoA-driven lipid synthesis. This may help prevent the long-term deleterious effects of excessive nutritional intake on liver mitochondrial function and insulin sensitivity, thereby preventing the development of metabolic diseases such as fatty liver and NAFLD (269). As described in the mechanism section, intermittent hypoxia leads to disturbances in the gut microbiota-circulating exosome pathway, disrupting adipocyte homeostasis and leading to metabolic dysfunction manifested as IR, whereas experiments have shown that such changes can be attenuated by physical activity, as regular non-strenuous activity will lead to substantial improvements in the gut microbiota-exosome pathway (270). In addition, available data suggest that aerobic exercise can lead to increased insulin sensitivity and enhanced glucose metabolism through a variety of different molecular mechanisms, including upregulation of insulin transporters on cell membranes of insulin-dependent cells, reduction of adipokines, normalization of redox status, improvement of β-cell function, regulation of IRS-1 phosphorylation, reduction of ceramide plasma levels, and induction of angiogenesis, which may lead to a reduced incidence of diabetic complications, as well as other metabolic effects (271, 272). Other forms of exercise, such as yoga, have also been shown to improve IR. Several meta-analyses have shown that yoga is a safe and effective intervention to reduce waist circumference and systolic blood pressure in patients with metabolic syndrome, particularly in improving cardio-metabolic health (273, 274). Some traditional Chinese health exercises, such as qigong and tai chi, have also been shown to have a measurable effect on weight, waist circumference, leg strength, increase HDL cholesterol, and result in significant improvements in IR (275, 276).

#### 5.1.2 Diet and nutrition therapy

As mentioned above, high-fat diets and the obesity they induce are a major cause of IR. Conversely, weight loss, when necessary, and dietary interventions such as intermittent fasting programs that reduce carbohydrates in the diet can significantly improve glycemic and insulin responses. From the available scientific data, reducing total daytime carbohydrate intake to 40-50% of daily energy intake, such as a Mediterranean-style diet and high protein diet, is one of the key dietary habits for improving IR (267). The Mediterranean diet is characterized by a wide range of cardio-protective nutrients, with beneficial effects on several outcomes related to metabolic health, and significant beneficial changes in metabolic risk factors, including HOMA-IR index (277–279). There are also RCT studies reporting that a high-protein diet is more effective in controlling IR and glycemic variability compared to a Mediterranean diet, which may be related to the satiety and increased metabolic rate associated with a high-protein, low-sugar diet (280).

In terms of dietary composition, a key dietary strategy for treating IR and improving glycemic control is to consume foods and meals that reduce the glucose fluctuations known to induce oxidative stress and beta cell damage (281). The contribution of high-fat diets to obesity and IR is well known. However, a single-minded approach to weight loss by replacing fat intake with carbohydrates is counterproductive because it could exacerbate IR. Researchers suggest that calorie restriction for weight loss and rationing of the macronutrient composition of the diet is important. What’s more, according to the suggestion from a recent meta-analysis, low-fat dairy intake has beneficial effects on abdominal adiposity and body weight, which may be associated with a reduced risk of IR and metabolic diseases (282, 283). The possible mechanism for this is that calcium and vitamin D in supplemental dairy products may facilitate lipolysis and optimize glucose metabolism (284). Carbohydrates are the main macro-nutrient influencing the glycemic response, especially after a meal. In recent years, some researchers have proposed that consumption of carbohydrates rich in dietary fiber and low glycemic index, such as whole grains, is beneficial in improving insulin sensitivity and metabolic flexibility, independent of gut hormones (285, 286). A recent meta-analysis reported that increasing daily fiber intake by 15 or 35 grams compared to a low-fiber diet reduced homeostatic model assessment of insulin resistance (HOMA-IR), leading to improvements in glycemic control, lipids, weight, and inflammation, as well as a reduction in premature mortality (287). Not only is the amount of carbohydrate intake important, but the timing of major carbohydrate intake during the day is also a determining factor in the increase in glucose and insulin after meals and the improvement or otherwise of IR (267). The results of some randomized controlled trial (RCT) studies suggest that it is advisable to consume at least half of the carbohydrates at lunch and to avoid consuming large amounts of carbohydrates at breakfast or dinner in order to control blood glucose spikes, which may be related to diurnal variations in insulin sensitivity (288–290). Results of another study showed that 10 hours of restrictive eating improved quality of life by reducing body weight and improving blood glucose, insulin sensitivity and related metabolic disorders (291). Other dietary strategies have been shown to prevent high-fat diet-induced IR, such as the intake of flavonoid-rich natural products, like flavonoids, which upregulate the expression of related genes through cell surface G protein-coupled estrogen receptors (292).

### 5.2 Pharmacological treatments

Although lifestyle modification and weight loss are highly recommended to improve IR and its associated metabolic disorders, they have limited effectiveness, slow onset of action, and low feasibility. Pharmacological treatments to increase insulin sensitivity will be described next.

#### 5.2.1 Chemotherapy

Currently, the main drugs that can effectively improve IR are anti-hyperglycemic drugs, including metformin, thiazolidinediones (TZD), sodium glucose cotransporter (SGLT)-2 inhibitors (SGLT2i), etc., which are listed in **Table 1** and will be described sequentially below.

**Table 1:** Clinically used antihyperglycemic drugs to improve IR.

Type	Listed drugs	Mechanisms	References
Biguanides	Metformin	Augmentation of peripheral glucose utilization by induction of GLUT4 expression and its translocation to the plasma membrane.	(293)
TZD	Pioglitazone, rosiglitazone	Enhancing insulin-mediated glucose uptake also inhibits the production of pro-inflammatory cytokines and triggers the release of adiponectin	(294)
SGLT2i	Canagliflozin, Dapagliflozin, Empagliflozin, tofogliflozin	Inhibits glucose reabsorption by the proximal renal tubule and improves insulin sensitivity by reducing body weight or glucose toxicity.	(295)
Sulfonylurea drugs	Glimepiride, glipizide	Promotes insulin receptor activation, thereby increasing the number of glucose transporter proteins and increasing insulin sensitivity	(296)
GLP-1 receptor agonists	Dulaglutide, Albiglutide, Liraglutide, Semaglutide	Reduces inflammation and oxidative stress and regulates lipid metabolism by increasing expression of glucose transporter proteins in insulin-dependent tissues	(297)

Metformin, the most commonly used insulin-sensitizing agent, has been a guideline-recommended first-line treatment for T2DM for decades and has recently found new applications in the prevention and treatment of various diseases, including metabolic disorders and cardiovascular diseases (298). A meta-analysis summarizing 31 RCT trials confirmed that treatment with metformin in populations at high risk for diabetes improved weight, lipid profile, and IR and resulted in a 40% reduction in new-onset diabetes (299). Metformin improves IR by modulating metabolic mechanisms and mitochondrial biogenesis through altering microRNAs levels by AMPK-dependent or AMPK-independent mechanisms (300). TZDs, such as pioglitazone, are potent insulin sensitizers targeting PPARγ and PI3K, regulating the transcription of nuclear transcription factors, stimulating mainly white adipose tissue remodeling, and regulating lipid flux for insulin sensitization and beta cell protection (294, 301). SGLT2i is a relatively new class of glucose-lowering drug that not only lowers blood glucose by inhibiting renal glucose reuptake, leading to increased urinary glucose excretion and lower blood glucose, but also improves insulin sensitivity in patients with T2DM by reducing body weight or glucose toxicity (302, 303). And in a randomized, double-blind, placebo-controlled clinical trial, it was shown that 8 weeks of treatment with SGLT2i empagliflozin restored insulin sensitivity in the hypothalamus of patients with prediabetes (304). Glucose-lowering drugs have also shown good, stacked effects in patients who do not have good response with one drug alone. For example, the addition of rosiglitazone to metformin can be clinically important in improving glycemic control, insulin sensitivity and beta-cell function (305). The addition of sitagliptin or metformin to pioglitazone monotherapy also leads to faster and better improvement in IR and inflammatory status parameters (306).

Other therapies, as well as some new drugs in clinical trials, such as anti-inflammatory drugs, drugs that target hepatic lipid and energy metabolism, renin-angiotensin-aldosterone system blockers, vitamin D, antioxidants, probiotics and fecal transplants, have also shown improvement in IR (220). Among them, selected clinical trials in the last decade have been listed in **Table 2**. As mentioned previously, low-grade chronic inflammation is associated with IR and metabolic disturbances. For example, in *in vitro* and *in vivo* mouse models of diet-induced hyperinsulinemia, low-dose naltrexone attenuates hyperinsulinemia-induced proinflammatory cytokine release and restores insulin sensitivity (307). However, it is worth noting that corticosteroids can cause IR and hyperglycemia due to their metabolic effects, and statins also increase the risk of IR, although they can reduce circulating inflammatory markers (220).

**Table 2:** Currently developing drugs targeting insulin resistance.

Targets	Starting time	Phase	Condition or disease	Intervention	Treatment schedule	Primary outcome measures	Secondary Endpoints:	Study design	NCT number
microbial gut	2020	Phase 4	Obesity Insulin Resistance	Dietary Supplement: Synbiotic (Rillus) Drug: Placebo	12weeks	HOMA-IR	Abundance-based Coverage Estimator (ACE) Index of Faecal Sample; Shannon Index of Faecal Sample	A multiple-arm, non-Randomized study	NCT04642482
PPAR-α/δ agonist	2011	Phase 2	Insulin Resistance Abdominal Obesity	Drug: GFT505 80mg Drug: Placebo	8-week treatment period, a 6-week wash out period, a second 8-week treatment period; hard gelatin capsules dosed at 20mg, oral administration, 4 capsules per day before breakfast	Glucose Infusion Rate (GIR)	Differences in Hepatic Glucose Production (HGP); Changes in HGP (Hepatic Glucose Production)	A Multicentre, Randomised, Single Blind, Placebo-Controlled, Cross Over Study.	NCT01271777
PPAR-γ agonist	2021	Phase 2	Prostate Cancer Insulin Resistance	Drug: Pioglitazone 30 mg Drug: placebo tablet	Subjects will self-administer a 30 mg pioglitazone oral tablet daily For patient taking a diabetic regimen of gemfibrozil, the dose will be 15 mg daily; 6 months.	insulin sensitivity	Insulin signaling	A prospective, randomized, double-blind, placebo-controlled trial	NCT04995978
Intestinal microbiota	2016	Phase 2	Obesity, Morbid Insulin Resistance	Biological: Fecal filtrate from 150 g stool from healthy lean donors Biological: Fecal filtrate from 150 g of the recipient’s own stool	150 g stool from healthy lean donors will be diluted in 0.9% normal saline to a total volume of 450 mL; 12 weeks	HOMA-IR	Body weight, BMI, %weight change, appetite score, quality of life questionaire, depression score, anxiety score	A randomized controlled trial	NCT02970877
NLRP3 inflammasome	2021	Phase 2	Obesity Insulin Resistance Inflammation	Drug: Colchicine Drug: Placebo	Colchicine 1 capsule (0.6 mg) per day; 12 weeks	HOMA-IR	Fasting serum insulin, fasting serum glucose, High-Sensitivity C-Reactive Protein, Matsuda Index	A randomized, Controlled Trial	NCT05017571
LDL receptor	2013	Phase 3	Metabolic Syndrome X	Drug: Ezetimibe Drug: Placebo	Ezetimibe 10 mg/d; 12 weeks	Change in Intestinal mRNA Expression Levels of LDL Receptor	Change in Intestinal mRNA Expression Levels of SREBP-2, NPC1L1, ABCG5/8, PCSK9 and HMG CoA; Change in Intestinal Protein Levels of LDL Receptor; Change in Protein Levels of SREBP-2, NPC1L1, ABCG5/8, PCSK9 and HMG CoA Reductase	Randomized; Crossover Assignment	NCT01849068
renal SGLT2	2015	Phase 4	Type 2 Diabetes Mellitus	Drug: Dapagliflozin Drug: Placebo	Dapagliflozin 10 mg Tablets, Oral, Once Daily, 8 weeks	Adjusted Change From Baseline in Skeletal Muscle Insulin-stimulated Gluocose Uptake	Adjusted Change in Adipose Tissue Insulin-stimulated Glucose Uptake; Adjusted Change in Liver Insulin-stimulated Glucose Uptake	single centre, randomized, parallel-group, double-blind, placebo-controlled Phase IV study	NCT02426541
PDE5 i	2015	Phase 2	Type 2 Diabetes Mellitus	Drug: Tadalafil Drug: Placebo	Per oral intake of tadalafil 20 mg o.d.; six weeks	insulin sensitivity (glucose disposal rate)	Mean glucose (HbA1c, mmol/mol) in blood; Fasting plasma glucose levels; Arginine-induced insulin secretion (area under curve, AUC, mU/l/min); Levels interstitial insulin; Lactate concentrations in insulin sensitive tissues; Levels of inflammatory markers in blood; Endothelial function in peripheral arteries measured with EndoPAT,	a double-blind, placebo-controlled crossover study	NCT02601989
PI3-kinase, Akt, GLUT-4, PPAR-γ, and PPARδ	2013	Phase 3	Polycystic Ovary Syndrome (PCOS) Insulin Resistance	Drug: DLBS3233 Drug: Metformin XR Drug: Placebo metformin Drug: Placebo DLBS3233	DLBS3233 100 mg capsule once daily; Metformin XR 750 mg caplet twice daily,	HOMA-IR	Lipid profile improvement; Improvement of glucose tolerance; Change of waist circumference; Response rate: presence of ovulation; Change of endometrium thickness; mprovement of S/A ratio; Improvement in Ferriman-Gallwey Score; Reduction of free testosterone level; Change of luteinizing hormone (LH) level; Change of luteinizing hormone (LH)/follicle stimulating hormone (FSH) ratio	3-arm, randomized, double-blind, double-dummy, and controlled clinical study	NCT01999686
angiotensin-receptor blocking (ARB)	2014	Phase 4	Essential Hypertension Complicated by Type 2 Diabetes Mellitus	Drug: Azilsartan Drug: Telmisartan	azilsartan 20 mg once daily in the morning; 12 weeks	Change in Insulin Resistance Index (HOMA-R)	Change in Fasting Blood Glucose; Change in Fasting Insulin; Change in Glycosylated Hemoglobin (HbA1c); Change in Homeostasis Model Assessment of Beta Cell Function (HOMA-β); Change in 1,5-anhydroglucitol (1,5-AG); Number of Participants With Treatment-Emergent Adverse Events	Multicenter, randomized, open-label, parallel-group exploratory study	NCT02079805
amino acid	2021	Not Applicable	Obesity Insulin Resistance	Dietary Supplement: Oral glutamine supplementation Dietary Supplement: Oral protein powder supplementation	oral glutamine supplementation 10 g Ter In Die; 8 weeks	HOMA-IR	Irritable Bowel Syndrome (IBS) severity score; feces consistency	a blinded randomized controlled trial	NCT04883515
bariatric procedures	2017	Not Applicable	Insulin Resistance Obesity	Procedure: Roux-en-Y Gastric Bypass (RYGBP) Procedure: Sleeve Gastrectomy (SG) Behavioral: Very Low Calorie Diet (VLCD)	standard RYGBP procedure; standard SG procedure	Change in urine free cortisol level		a non-randomized prospective study	NCT03371368
lifestyle intervention	2022	Not Applicable	Diabetes Mellitus, Type 2 Insulin Resistance	Procedure: Passive heating Procedure: Thermoneutral	perform baths in 38°C natural thermal mineral water a maximum of five times per week; 12 weeks	Change in hemoglobin A1c level	Change in fasting plasma glucose; Change in fasting insulin; HOMA-IR; Decrease of daily insulin dose; BMI; Change in mean blood pressure; Change in heart output; Change in the prevalence of electrocardiogram events; Change in the proportion of hypertension, retinopathy, nephropathy, neuropathy; Change in total cholesterol level, LDL level, HDL level, triglyeride level, ALP, ALT, AST, GGT, glomerular filtration rate, creatinine level, etc.	a two-arm, randomized, controlled study	NCT05237219
antioxidation	2020	Not Applicable	Non-Alcoholic Fatty Liver Disease Insulin Resistance	Drug: Nutraceutical therapy	Oral administration of 303mg of silybin-phospholipid complex, 10mg of vitamin D, and 15mg of vitamin E, twice a day; six months	HOMA-IR	Mean change in ALT levels, insulin levels, CRP levels, TBARS levels	randomized; open label, parallel assignment	NCT04640324
electrical stimulation	2019	Not Applicable	Obesity Overweight Insulin Resistance	Device: Neuromuscular Electrical Stimulation (Sensory) Device: Neuromuscular Electrical Stimulation Other: Resistance Training	Group will receive Electrical Stimulation up to maximum tolerable level (30min/day, 3x/week); 8 weeks	Glycemic Control	Respiratory Exchange Ratio; Amount of lean mass	Randomized; Parallel Assignment	NCT03947697

#### 5.2.2 Traditional Chinese medicine treatment

TCM plays an equally critical role in the treatment of many acute and chronic diseases, especially its adeptness in restoring the dynamic balance of the body in systemic diseases. Its main therapeutic measures include herbal medicine, acupuncture and Tui Na. Several classical herbal formulations have been widely used in the clinical treatment of T2DM and various other metabolic disorders. For example, GegenQinlian decoction improves IR in fat, liver and muscle tissue through a variety of compounds, targets, pathways and mechanisms (308). Yi Qi Zeng Min Tang has been shown to improve IR in high-fat fed Sprague-Dawley rats without increasing body weight (309). Because it reduced the expression of PI3K p85 mRNA and IRS1 protein, Fu Fang Zhen Zhu Tiao Zhi formula similarly improved IR *in vitro* and in rats with metabolic syndrome (310).Gui Zhi Fu Ling Wan, Dingkun Pill and Liuwei Dihuang Pills are herbal formulas widely used in the treatment of gynecological disorders and have the effect of harmonizing Qi and blood or dispelling blood stasis in Chinese medical theory. In modern clinical and animal studies, they have been found to be very effective in the treatment of PCOS and also slightly improve IR by alleviating inflammation, remodeling intestinal homeostasis, or by regulating the PI3K/Akt signaling pathway, among other mechanisms (311–314). In addition, the efficacy of acupuncture in improving IR is equally impressive, as a recent meta-analysis showed that acupuncture improved HOMA-IR and ISI as well as fasting blood glucose (FBG), 2h postprandial blood glucose (2hPG) and fasting insulin (FINS) levels, with fewer adverse events (315).

## 6 Conclusion and perspective

The increased incidence of IR and its vital role as a major and common cause of numerous metabolic diseases have created an urgent need to gain insight into the etiology and pathogenesis of IR, as well as to explore better early diagnostic methods and therapeutic strategies for it. The diagnosis of insulin resistance is currently inconclusive, while it is important to detect IR early and predict individual response to treatment. In addition to the few simple indices of IR calculated from biochemical or anthropometric variables currently in use, emerging biomarkers may now be the way forward, but this still needs to be supported by clinical data. Different ranges and criteria are also needed for the diagnosis and monitoring of different metabolic diseases. As mentioned above, IR is a central mechanism in many metabolic diseases. Since this is the case, IR should be considered as a therapeutic target for patients with a combination of multiple metabolic diseases so that multiple diseases can be treated simultaneously with the same treatment approach, thereby reducing healthcare expenditures. Although there is no universally accepted theory to explain the mechanisms that cause IR. Nevertheless, there is growing evidence linking ectopic lipid accumulation, ER stress, plasma concentration of inflammatory cytokines, oxidative stress, abnormalities in insulin signaling, and other factors to IR. In recent years, the exploration of the molecular mechanisms of IR has also led to the emergence of new therapeutic concepts beyond metformin and TZD. Regardless lifestyle modification remains the most basic and least costly intervention. Normative criteria need to be developed for different metabolic diseases considering IR as a focus.

## Author contributions

FL and HJ provided the idea of the manuscript. XZ, XA, and CY contributed equally to this manuscript. XZ, XA, WS, and CY drafted the manuscript and searched the relevant literature. XZ and XA drafted the figures, and all authors approved the final version of the manuscript. All authors agree to be accountable for all aspects of work ensuring integrity and accuracy. All authors contributed to the article and approved the submitted version.
